# What Do Human Parasites Do with a Chloroplast Anyway?

**DOI:** 10.1371/journal.pbio.1001137

**Published:** 2011-08-30

**Authors:** Sethu C. Nair, Boris Striepen

**Affiliations:** 1 Department of Cellular Biology, University of Georgia, Athens, Georgia, United States of America; 2 Center for Tropical and Emerging Global Diseases, University of Georgia, Athens, Georgia, United States of America

## Abstract

The malaria parasite has a chloroplast, which is a holdover from its algal past. The authors discuss the fascinating biology of this organelle and its promise for treatment in light of a seminal new study.

Apicomplexans are an important group of pathogens that include the causative agents of malaria, toxoplasmosis, and cryptosporidiosis. These single-celled eukaryotic parasites evolved from photosynthetic algae. A remnant chloroplast, called the apicoplast, is a telltale hold-over from this more benign past in the ocean. The apicoplast is essential for parasite growth and development and therefore a potential target for drug therapy. The fact that humans and animals lack chloroplasts suggests that using approaches to target the apicoplast may provide parasite specificity. What are the critical functions of the apicoplast that should be targeted? In addition to the obvious medical relevance this question has broader biological implications. Why do organisms maintain an ancient symbiotic relationship when the initial rationale for this relationship has fallen by the evolutionary wayside? A new study by Yeh and DeRisi provides important clues. Their work demonstrates that antibiotic-induced loss of the apicoplast in cultured malaria parasites can be chemically rescued by providing isopentenyl-pyrophosphate (IPP) in the medium. IPP is generated by the apicoplast resident isoprenoid biosynthesis pathway and is apparently the one apicoplast metabolite that the parasite cannot live without in the red blood cell. This finding could be of great importance for the development of drugs and vaccines. The ability to produce and maintain parasite lines that lack the apicoplast also offers exciting experimental possibilities for the future.

## Apicomplexan Parasites, the Dark Side of the Algal World

Apicomplexans are a phylum of protists that live as intracellular parasites in a tremendous variety of vertebrate and invertebrate animals. A number of apicomplexans cause serious disease in humans. These include, importantly, five species of the genus *Plasmodium*, the causative agents of malaria. Malaria remains one of the most devastating infectious diseases, with a staggering death toll in particular among small children in sub-Saharan Africa. *Toxoplasma* and *Cryptosporidium* are apicomplexans that cause fetal and early child hood diseases worldwide, and they are of particular concern for individuals with a weakened immune system such as those with HIV-AIDS. Lastly, several apicomplexans, such as *Theileria*, *Babesia*, or *Eimeria*, threaten the health of domestic animals and thus indirectly impinge on human health and prosperity.

**Figure 1:**
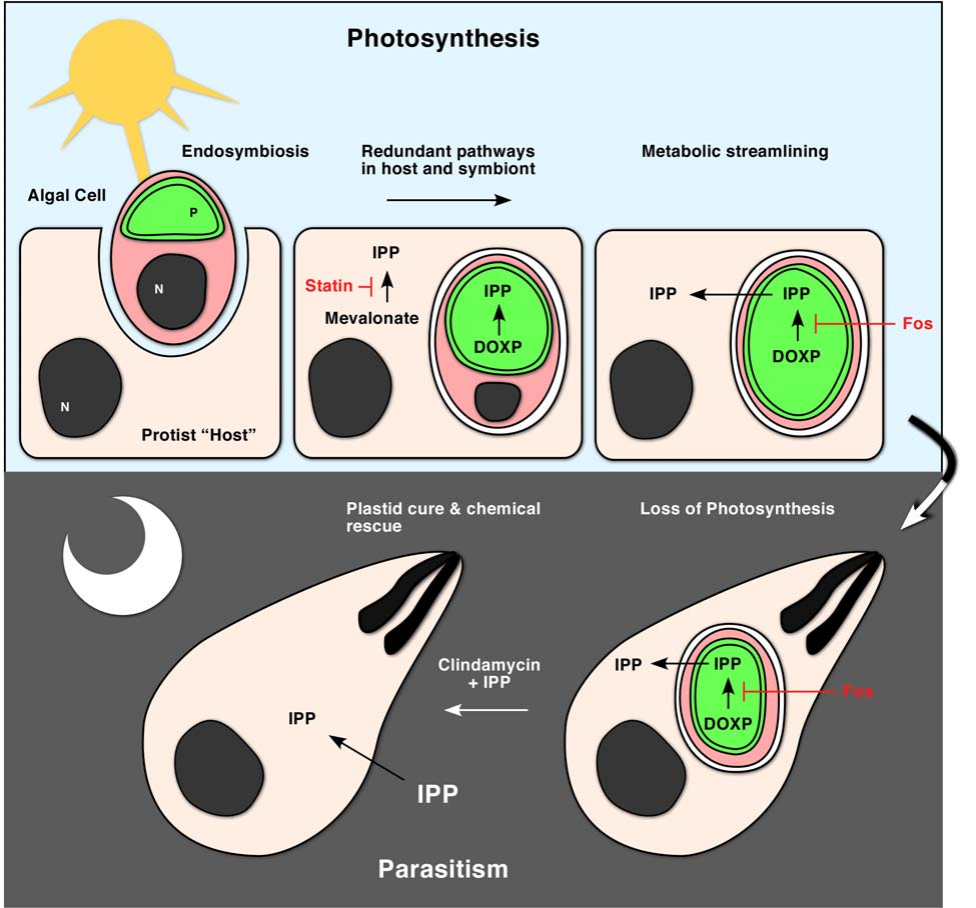
A metabolic Catch-22 due to evolutionary acquired isoprenoid dependency. Schematic representation of the putative evolution of the apicoplast and its metabolism. The ancestor of apicomplexans arose from the endosymbiotic merger of a protist host and a single celled red alga. The host likely benefited from the symbiont's ability to photosynthesize. Initially there were two redundant isoprenoid synthesis pathways: a cytoplasmic pathway (likely using the mevalonate pathway as ciliates still do [47]) and a plastid DOXP pathway. Note that both pathways have multiple enzymatic steps and are shown highly simplified here. The cytoplasmic pathway was lost producing dependency. This forced maintenance of the apicoplast even after loss of photosynthesis (lower panel). High concentrations of exogenously supplied IPP can overcome this dependency and thus rescue clindamycin induced loss of the organelle [31] (clindamycin specifically blocks apicoplast protein synthesis). Fos, fosmidomycin; N, nucleus; P, plastid; Statin, the active principle of the cholesterol-lowering drug Lipitor).

Apicomplexans evolved from a photosynthetic ancestor, a finding that initially took the field of parasitology research by surprise [1]–[4]. They are part of a large branch of the eukaryotic tree of life that is now known as the chromalveolates [5]. This group came into the world through the merger of two eukaryotic cells: a protist host and a red algal endosymbiont (Figure 1). Over time the endosymbiont alga was transformed into the current chloroplast-like organelle [6]. Many of the chromalveolate kin of the apicomplexans are algae that harvest sunlight by photosynthesis, including kelps, diatoms, dinoflagellates, and haptophytes. Collectively they are responsible for the bulk of primary production and carbon fixation in the ocean. Others, such as ciliates and oomycetes, have lost their plastids [7]. Apicomplexans are unique in that they largely maintained the plastid despite the fact that they are no longer photosynthetic (a few, such as *Cryptosporidium*, have lost the organelle [8],[9]).

## Why Did the Parasites Maintain an Apparently Pointless Organelle?

A metabolic Catch-22 may mean that these parasites cannot afford to lose it: A series of pharmacological and genetic studies in *Toxoplasma* and *Plasmodium* has consistently demonstrated that the apicoplast is essential for parasite growth [10]–[12]. Chloroplasts in plants have metabolic functions beyond photosynthesis and provide the cell with a number of crucial compounds. Genomic and experimental analyses in apicomplexans suggest that these nonphotosynthetic functions of the chloroplast are largely conserved in the apicoplast. The apicoplast is home to biosynthetic pathways for fatty acids, isoprenoids, iron sulfur cluster assembly, and a segment of the heme pathway [13],[14]. Several of these compounds are metabolically expensive—they require large amounts of energy and reduction power to synthesize. Harnessing the power of photosynthesis directly at its source in the chloroplast to generate such expensive compounds makes metabolic sense. The host took advantage of this economy and, through loss of redundant cytoplasmic pathways, came to rely on the symbiont. When apicomplexans abandoned photosynthesis instead of producing metabolites for free, the apicoplast had to be fed with energy and carbon precursors from the cytoplasm of the parasite [15],[16]. Not only do the pathways have to be fed, but the entire symbiont, with its elaborate inheritance, gene expression, protein import, and replication machinery, has to be maintained [6],[17],[18]. Which of the metabolic functions of the apicoplast are truly essential and thus the reason for its persistence at considerable cost? This is not an entirely academic question as the apicoplast is a hotly pursued drug target [19]. This is of particular importance for malaria, in which drug resistance develops rapidly [20], requiring a continuous pipeline of novel antiparasitic compounds. Recent discussion of a renewed effort towards the goal of malaria elimination will likely require an even deeper portfolio of drugs [21].

## To IPP or Not To Be, That Is the Function

Two biosynthetic pathways known for their importance in the biology of the plant chloroplast emerged as candidates for the most critical apicoplast function(s): the synthesis of fatty acids and isoprenoid precursors. Importantly, both pathways are of cyanobacterial origin (the bacterial ancestor of all plastids) and are different from those in their mammalian counterparts [22],[23]. At least in the malaria parasite both pathways appeared to be the only *de novo* synthesis machine for these important metabolites, and antibiotics thought to selectively interfere with the plastid type pathway inhibited parasite growth [23]–[25]. Subsequent genetic studies showed that loss of apicoplast fatty acid synthesis is lethal in *Toxoplasma* and the liver stages of *Plasmodium*, but that it can be tolerated in the clinically most important red blood cell (RBC) phase of malaria [26]–[28]. These findings suggested that in the RBC the parasite can salvage fatty acids from its host. This left the isoprenoid pathway as the putative *raison d'etre* for the apicoplast, and a series of recent papers now provide robust support for the importance of this pathway for the parasite [29]–[31].

Isoprenoids are a large class of biological compounds that include natural rubbers, cholesterol, ubiquinone, dolichol, farnesol, and many others. This tremendous diversity is derived from a simple five-carbon precursor isopentenyl pyrophosphate (IPP) and its isomeric form dimethylallyl pyrophosphate (DMAPP). Nature has devised two mechanistically distinct synthesis routes for IPP that are named after their key intermediate mevalonate and 1-deoxy-D-xylulose-5-phosphate (DOXP) pathways. Animals and fungi use the mevalonate pathway (the target of the blockbuster drug atorvastatin [Lipitor]), while most bacteria and plants rely on the DOXP pathway. Apicomplexan parasites depend on their apicoplast-localized DOXP pathway. The antibiotic fosmidomycin, a specific inhibitor of DOXP reductoisomerase, blocks the growth of malaria parasites in culture and in animal experiments [23], and mutations in the DOXP pathway are lethal in *Toxoplasma* [29]. This demonstrates clearly that the DOXP pathway is important, but is it the only important function? Yeh and DeRisi have put this question to an experimental test that is truly elegant in its simplicity and publish their results in this issue of *PLoS Biology* [31]. In experiments in cultured *P. falciparum* they demonstrate that parasites can be rescued from the lethal effects of fosmidomycin treatment when IPP, the final product of the target pathway, is provided in the medium. This chemical complementation occurs only with IPP, requires a substantial dose (200 µM), and is not observed for DMAPP or the alcohol form of IPP, isopentanol. Next they tested whether IPP rescue also extends to parasites that are treated with antibiotics that more generally impede the apicoplast by inhibiting its protein synthesis, such as clindamycin, doxycycline, or chloramphenicol. Not only does IPP rescue this treatment, but fascinatingly this apparently cures parasites of their plastid organelle. When parasites are grown in the continuous presence of IPP and antibiotic, the organelle and its genome are lost. These plastid-less strains now depend on a continuous exogenous supply of IPP, and withdrawal results in swift death. This seminal finding suggests that the apicoplast and the roughly 500 proteins that work there [32] are ultimately maintained for a single purpose: the synthesis of a simple isoprenoid precursor. Note that this is true for the *P. falciparum* in RBCs and culture medium and that additional functions are required in other phases of the life cycle, e.g., in the liver [28].

## The Apicoplast and Its Potential for Drugs and Vaccines

Overall, recent work suggests the DOXP pathway as a prime target for the development of new antiparasitic drugs. Indeed, fosmidomycin is effective in the treatment of malaria in people when combined with a second antibiotic that shows synergism in targeting the apicoplast [33]. Surprisingly, however, the drug has no effect on most other apicomplexans [34],[35]. While the target enzyme of fosmidomycin, e.g., in *Toxoplasma*, appears essential and drug sensitive, the parasite is nonetheless resistant to concentrations many hundred-fold higher than those needed to inhibit *Plasmodium* [29],[36]. The key to this conundrum lies in drug access. While Baumeister and colleagues proposed a model in which the host cell is the main barrier to drug access [36], we favor the parasite membrane as the main impediment. In support of this hypothesis we genetically engineered a parasite capable of drug uptake, which renders a resistant parasite line fully susceptible in the absence of any changes in the host cell [29]. Lack of uptake is similarly responsible for fosmidomycin resistance in *Mycobacterium tuberculosis* [37]. At any rate, both studies point to the need for active drug uptake as an Achilles' heel of fosmidomycin. A mutation in the proteins that transport the drug into the parasite cell or into the apicoplast could rapidly confer drug resistance. The long-term mainstay of malaria treatment chloroquine fell to resistance mutations in such a transporter, the food vacuole protein PfCrt [38]. Eliminating charge from fosmidomycin to enhance uptake without compromising activity has been challenging [39], but some moderate enhancement has been achieved using various ester modifications [40]. The discovery of novel DOXP pathway inhibitors with more favorable uptake and pharmacokinetic characteristics is a high priority, and may produce drugs not only for parasites but also for bacterial disease such as tuberculosis [41].

Does the finding that IPP is the most critical product of the apicoplast by Yeh and DeRisi [31] mean that the isoprenoid pathway is the only apicoplast target worth pursuing? While targeting the isoprenoid pathway may provide the most direct and potentially fastest impact, it's not the only target. Antibiotics that interfere with apicoplast protein translation are already in clinical use for malaria and toxoplasmosis [42]. Targeting other aspects of apicoplast maintenance or metabolism that would ultimately interfere with the ability of the organelle to supply IPP is a viable approach that has not been fully explored. We also do not yet understand what the final isoprenoid product is for which the parasite so dearly requires IPP. A number of candidates would link the plastid to mitochondrial function, hormone action, or protein modification [43],[44]. Some of these may be excellent targets. Exploring these many functions of the apicoplast and its downstream metabolism is likely a more straightforward task now that parasites that lack the organelle can be maintained by chemical complementation [31]. This offers a number of metabolomic, proteomic, and genetic routes to more fully dissect apicoplast biology and function. Generating plastid-minus parasites potentially could also be a way to produce live attenuated vaccines. Genetically attenuated parasites have shown some promise [45]. Recent studies used drugs that target apicoplast functions to treat early infection and, intriguingly, this treatment not only effected cure but also generated immunological protection from reinfection [46]. The new plastid-minus mutant displays an inherent growth limitation in the absence of IPP that may act similar to this pharmacological attenuation. Limited growth is desirable in a vaccine strain. In contrast to dead parasites, attenuated organisms like the plastid-minus parasites enter their host cells and initiate development (yet ultimately succumb to their lack of IPP). This may stimulate the immune response in a more natural and sustained fashion. Although the apicoplast provides tremendous opportunities as a target, we have only begun to understand and exploit its complex biology. Studying plastidless parasites undoubtedly will be highly informative.

## Abbreviations

DMAPP: dimethylallyl pyrophosphate
DOXP: 1-deoxy-D-xylulose-5-phosphate
IPP: isopentenyl pyrophosphate
RBC: red blood cell
